# *NNAlign*: A Web-Based Prediction Method Allowing Non-Expert End-User Discovery of Sequence Motifs in Quantitative Peptide Data

**DOI:** 10.1371/journal.pone.0026781

**Published:** 2011-11-02

**Authors:** Massimo Andreatta, Claus Schafer-Nielsen, Ole Lund, Søren Buus, Morten Nielsen

**Affiliations:** 1 Center for Biological Sequence Analysis, Technical University of Denmark, Kongens Lyngby, Denmark; 2 Schafer-N, Copenhagen, Denmark; 3 Laboratory of Experimental Immunology, Faculty of Health Sciences, University of Copenhagen, Copenhagen, Denmark; University College Dublin, Ireland

## Abstract

Recent advances in high-throughput technologies have made it possible to generate both gene and protein sequence data at an unprecedented rate and scale thereby enabling entirely new “omics”-based approaches towards the analysis of complex biological processes. However, the amount and complexity of data that even a single experiment can produce seriously challenges researchers with limited bioinformatics expertise, who need to handle, analyze and interpret the data before it can be understood in a biological context. Thus, there is an unmet need for tools allowing non-bioinformatics users to interpret large data sets. We have recently developed a method, *NNAlign*, which is generally applicable to any biological problem where quantitative peptide data is available. This method efficiently identifies underlying sequence patterns by simultaneously aligning peptide sequences and identifying motifs associated with quantitative readouts. Here, we provide a web-based implementation of *NNAlign* allowing non-expert end-users to submit their data (optionally adjusting method parameters), and in return receive a trained method (including a visual representation of the identified motif) that subsequently can be used as prediction method and applied to unknown proteins/peptides. We have successfully applied this method to several different data sets including peptide microarray-derived sets containing more than 100,000 data points.

*NNAlign* is available online at http://www.cbs.dtu.dk/services/NNAlign.

## Introduction

Proteins are extremely variable, flexible and pliable building blocks of life that are crucially involved in almost all biological processes. Many diseases are caused by protein aberrations, and proteins are frequent targets of intervention. A plethora of high-throughput methods are currently being used to study genetic associations and protein interactions, and intense on-going international efforts aim at understanding the structures, functions and molecular interactions of all proteins of organisms of interest (e.g. the Human Proteome Project, HPP). In some cases, linear peptides can emulate functional and/or structural aspects of a target structure. Such peptides are currently identified using simple peptide libraries of a few hundreds to thousands peptides whose sequences have been systematically derived from the target structure at hand – that is, if this is known. Even when the native target structure is unknown, or too complex (e.g. discontinuous) to be represented by homologous peptides, the enormous diversity and plasticity of peptides may allow one or more peptides to mimic relevant aspects of a given target structure [1], [2].

Peptides are therefore of considerable biological interest and so are methods aimed at identifying and understanding peptide sequence motifs associated with biological processes in health and disease. Indeed, recent developments in large-scale, high-density peptide microarray technologies allow the parallel detection of thousands of sequences in a single experiment, and have been used in a wide range of applications, including antibody-antigen interactions, peptide-MHC interactions, substrate profiling, identification of modification sites (e.g. phosphorylation sites), and other peptide-ligand interactions [3], [4], [5], [6], [7]. One of the major advances of peptide microarrays is the ease of generating large numbers of potential target structures and systematic variants hereof [8].

Given the capability for large-scale data-generation already realized in current “omics” and peptide microarray-based approaches, experimentalists will increasingly be confronted with extraordinary large data sets and the consequent problem of identifying and characterizing features common to subsets of the data. These are by no means trivial problems. Up to a certain level of size and complexity, data can be presented in simple tabular forms or in charts, however, larger and/or more complex bodies of data (e.g. in proteome databases) will need to be fed into bioinformatics data mining systems that can be used for automated interpretation and validation of the results, and eventually for *in silico* mapping of peptide targets. Moreover, such systems can conveniently be used to design next-generation experiments aimed at extending the description of target structures identified in previous analyses [9].

A wealth of methods has been developed to interpret quantitative peptide sequence data representing specific biological problems. By way of examples, SignalP, which identifies the presence of signal peptidase I cleavage sites, is a popular method for the prediction of signal peptides [10]; LipoP, which identifies peptidase II cleavage sites, predicts lipoprotein signal peptides in Gram-negative bacteria [11]; various prediction methods predict phosphorylation sites by identifying short amino acid sequence motifs surrounding a suitable acceptor residue [12], [13], [14], [15] etc. In general terms, these methods can be divided in two major groups depending on the structural properties of the biological receptor investigated, and of the nature of the peptides recognized. The simplest situation deals with interactions where a receptor binds peptides that are in register and of a known length. In this case, the peptide data is pre-aligned, and conventional fixed length, alignment-free pattern recognition methods like position specific weight matrices (PSSM), artificial neural networks (ANN), and support vector machines (SVM) can be used. Peptide-MHC class I binding is a prominent example of the successful use of such methods to characterize receptor-ligand interaction represented by pre-aligned data (reviewed in [16]). Another more complex type of problems deals with interactions where either the motif lengths, and/or the binding registers, are unknown. In these cases, the peptide data must *a priori* be assumed to be unaligned and any bioinformatics method dealing with such data is faced with the challenge of simultaneously recognizing the binding register (i.e. performing an alignment) and identifying the binding motif (i.e. performing a specificity analysis). Peptide-MHC class II binding is a preeminent example of a receptor-ligand interaction represented by unaligned data. Several bioinformatics methods have been developed to identify binding motifs in such peptide data including Gibbs sampling [17], hidden Markov models (HMM) [18], stabilization matrix method (SMM) alignment [19], and alignment using artificial neural networks [20] (for more references see [21]). Another example of unaligned peptide data is that of antibodies interacting with linear peptide epitopes. Although B-cell epitopes frequently are conformational and three-dimensional in structure, some do contain linear components that can be represented by peptide interaction with the corresponding antibodies [22], [23], [24].

Even though most of the methods described above are standard methods for data-driven pattern recognition, the development of a prediction method for any given biological problem is far from straightforward, and the non-expert user will rarely be able to develop their own state-of-the-art prediction methods. We have recently described a neural network-based data driven method, *NN-align*, which has been specifically designed to automatically capture motifs hidden in unaligned peptide data [20]. *NN-align* is implemented as a conventional feed-forward neural network and consists of a two-step procedure that simultaneously identifies the optimal peptide-binding core, and the optimal configuration of the network weights (i.e. the motif). This method is therefore inherently designed to deal with unaligned peptide data, and it identifies a core of consecutive amino acids within the peptide sequences that constitute an informative motif. Note that the method does not allow for gaps in the alignment. Although *NN-align* was originally developed with the unaligned nature of peptide-MHC class II interaction in mind – and independent validations have shown that *NN-align* indeed performs significantly better than any previously published methods for MHC class II motif recognition [25] – the unique ability of this method to capture subtle linear sequence motifs in quantitative peptide-based data and its adaptability makes it extremely attractive for other applications as well. Here, we have adapted and extended the *NN-align* method so that it can handle quantitative peptide-based data in general. Making this method generally available for the scientific community, we have embedded it into a public online web-interface that facilitates both handling of input data, optimization of essential training parameters, visual interpretation of the results, and the option of using the resulting method to predict on user-specified proteins/peptides. Through the server the user can easily set up a cross-validation experiment to estimate the predictive performance of the trained method, and automatically reduce redundancy in the data. The logo visualization is also improved with an algorithm that aligns individual neural networks to maximize the information content of the combined alignment. This web-based extension of the *NN-align* method empowers experimentalists of limited bioinformatics background with the ability to perform advanced bioinformatics-driven analysis of his/her own sets of large-scale data.

## Results

Enabling any non-expert end-user to extract specific information from quantitative peptide data using an advanced bioinformatics approach, we have used our recently published *NN-align* method to generate a web-based extension with a reasonably simple, yet adaptable, web-interface and made this server publicly available at http://www.cbs.dtu.dk/services/NNAlign. Using this web server any user can submit quantitative peptide data (optimally based on actual discrete measurements, but even assigned classification, e.g. 0 and 1, can be used) and in return receive a trained method including training details and estimated predictive performance, a visual interpretation of the identified peptide pattern, and the trained model itself. The latter can be re-submitted to the web server at any later time and used to predict the occurrence of the learned motif in one or more concurrently submitted peptide sequences or FASTA format sequences.

The truly non-expert user has the option of using a set of default settings. Using these settings, the data is preprocessed using a linear transformation to make the data fall in the range from 0 to 1, and the *NN-align* method is trained using five-fold cross-validation. For each cross validation partition five networks, each initiated from different initial configurations, are trained with 3 hidden neurons. The only critical parameter that the user is required to specify is the motif length. The value used for this parameter is specific to each problem and the user is recommended to define a motif length (or an interval of motif length) that is relevant to the biological problem investigated by the peptide data. The default settings will in most cases allow the user to obtain a first impression of the motif contained in the data, and achieve a prediction method that allows the user to make prospective studies on uncharacterized proteins/peptides. The more experienced user has several advanced options to customize the training. For details on these options refer to Materials and Methods section, or the help section of the web-server.

**Figure 1:**
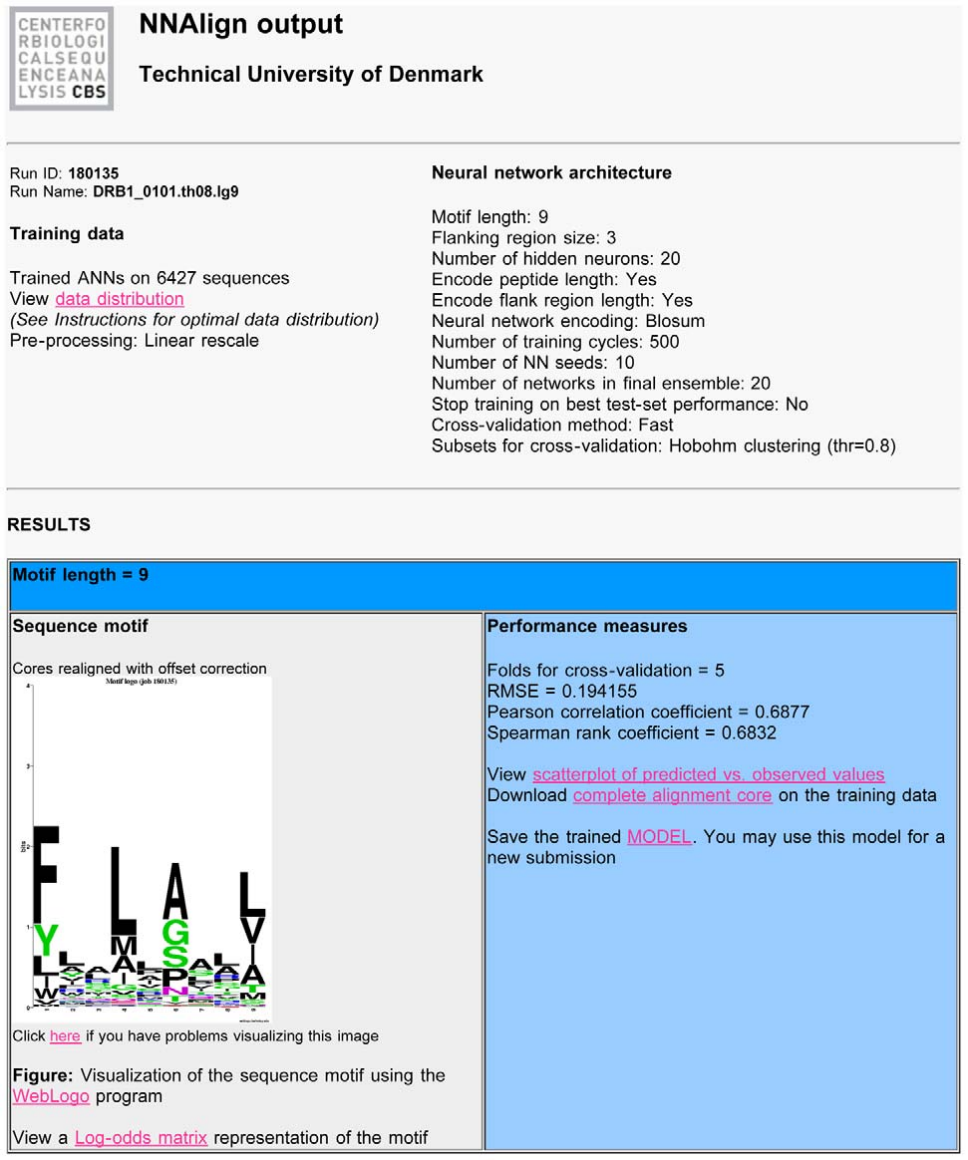
Example of output from the *NNAlign* server trained on MHC class II binding data for allele HLA-DRB1*0101. Links on the results page (in pink) redirect to additional files and figures relevant for the analysis. Run ID is a sequential identifier for the current job, and Run Name a user-defined prefix that is added to all files of the run. The “view data distribution” link shows the transformation applied to the data in pre-processing, which can be either a linear or logarithmic transformation. In this case the method was trained with a motif length of 9, including a PFR of size 3 to both ends of the peptide, and encoding in the network input layer peptide length and PFR length. The hidden layer was made of a fixed number of 20 neurons. Peptides were presented to the networks using a Blosum encoding to account for amino acid similarity, for 500 hundred iterations per peptide without stopping on the best test set performance. At each cross-validation step, 10 networks were trained starting from 10 different initial configurations. The subsets for cross-validation were constructed using a Hobohm1 method that groups in the same subset sequences that align with more than 80% identity (thr = 0.8). The model can be downloaded to disk using the dedicated link, and can be resubmitted to *NNAlign* to find occurrences of the learned pattern in new data. The estimated performance of the trained method is expressed in terms of Root Mean Square Error, Pearson and Spearman correlation. A visual representation of the correlation can be obtained from the scatterplot of predicted versus observed values. The “complete alignment core” link allows downloading the prediction values in cross-validation for each peptide, and where the core was placed within the peptides. Next follows a section on the sequence logo, showing a logo representation of the binding motif learned by the network ensemble. If the relative option is selected, links to logos for the individual networks in the final ensemble are also listed here. Finally, if an evaluation set is uploaded, an additional section shows performance measures and core alignment for these data.

An example output from the *NNAlign* Server is shown in Figure 1. Information about the training data is accompanied by a plot of the data distribution before and after the data processing needed to train the neural networks. An important feature is the possibility to download and save the trained model, and use it subsequently for predictions on new data. The results page also returns the performance of the method as estimated by cross-validation, and provides links to a scatter-plot showing the correlation between measured and predicted values, as well as the complete alignment core on the training data. A sequence logo gives a visual representation of the identified sequence motif, which can also be viewed in a log-odds position-specific scoring matrix format. If any evaluation data has been provided at the time of method training, a section of the results will report the predictions of this evaluation set.

A few example applications illustrating the power of the *NNAlign* method are presented in the following sections. First, the method is applied to examples of pre-aligned peptide data using examples of MHC class I binding. Next, the alignment problem is included using MHC class II binding data, showing the ability of the method to identify at the same time the correct length of the motif, the binding register, and the sequence motif itself. An important output from the *NNAlign* method is a sequence logo representing the identified binding motif. Such sequence logos provide a highly intuitive representation of single-receptor specificities (as is the case for MHC class I and II binding data). Finally, to illustrate how the method is capable of handling and guide the semi-expert user in interpreting large-scale data sets, *NNAlign* is applied to data generated by a large-scale peptide microarray technology.

### MHC class I

Binding of peptides to MHC class I molecules is highly specific, with only 1–5% of a set of random natural peptides binding to any given MHC molecule [26]. Moreover, in the vast majority of cases only peptides with length 8–10 amino acids can fit in the binding pocket of MHC class I molecules. The predictive performance of *NNAlign* on 12 human MHC class I alleles from data by Peters et al. [27] is shown in *Table 1* (see the table footnote for the parameters used). The benchmark data sets contain quantitative binding data of a given length (9 amino acids) covering the whole spectrum from non-binding to strong-binding peptides, hence serving as a perfect illustration of the strength of the *NNAlign* method to handle pre-aligned peptide data. The overall performances of the three methods are comparable demonstrating that *NNAlign* competes with state-of-the-art methods designed specifically for MHC class I prediction.

**Table 1:** Predictive performance in AUC on 12 human HLA MHC class I alleles (Peters data set) and on 14 HLA-DR MHC class II alleles (Wang similarity reduced SR dataset).

MHC class I					MHC class II					
ALLELE	#	*SMM*	*ANN*	*NNAlign*	ALLELE	#	*NN-align*	*SMM-align*	*Propred*	*NNAlign* server
A*0101	1157	0.980	**0.982**	0.980	DRB1*0101	3504	0.763	0.756	0.692	**0.794**
A*0201	3089	0.952	0.957	**0.959**	DRB1*0301	1136	0.829	0.808	0.669	0.816
A*0203	1443	0.916	0.921	**0.922**	DRB1*0401	1221	0.734	0.721	0.711	**0.736**
A*2402	197	0.780	**0.825**	0.772	DRB1*0404	474	**0.803**	0.789	0.753	0.782
A*0301	2094	0.940	0.937	**0.941**	DRB1*0405	1049	0.794	0.767	0.742	**0.808**
A*1101	1985	0.948	0.951	**0.952**	DRB1*0701	1175	0.811	0.796	0.75	**0.845**
A*2902	160	0.911	**0.935**	0.920	DRB1*0802	1017	0.698	0.689	0.641	**0.714**
A*3101	1869	0.930	0.928	**0.931**	DRB1*0901	1042	0.713	0.696		**0.745**
A*6801	1141	**0.885**	0.883	0.881	DRB1*1101	1204	0.847	0.829	0.779	**0.853**
B*0702	1262	0.964	**0.965**	0.961	DRB1*1302	1070	0.732	0.754	0.577	**0.775**
B*3501	736	**0.889**	0.875	0.876	DRB1*1501	1171	0.756	0.741	0.703	**0.765**
B*5301	254	0.882	**0.899**	0.875	DRB3*0101	987	**0.798**	0.78		0.784
Ave		0.914	0.922	0.914	DRB4*0101	1011	0.789	0.762		**0.808**
					DRB5*0101	1198	0.795	0.776	0.711	**0.798**
					Ave		0.776	0.762	0.703	0.787

For MHC class I no significant difference is found in predicted performance between the *NNAlign*, *SMM* and *ANN* method (p>0.5, binomial test). The values for the *SMM* and *ANN* methods were taken from Peters et al. [27]. The method was trained using a fixed motif length of 9 corresponding to the peptide length, and constructing a network ensemble with multiple architectures using respectively 2,3,4,5 and 7 hidden neurons. Performance was measured in cross-validation, training each network for a fixed number of 500 iterations per sequence.

The different MHC class II prediction methods are *NN-align* [20], *SMM-align* [19], and *Propred* [31], [32]. *NNAlign* server is the method described here. Performance values for first 4 methods are taken from [25]. *NNAlign* was trained with a motif length of 9, flanking regions of 3 amino acids, Blosum encoding including peptide length and flanking region length, and an ensemble of 2, 3, 5, 9 and 12 hidden neurons for each of 10 initial random configurations.

In bold is highlighted the best performing method for each MHC allele. The column # gives the number of the peptides in the data set for the given allele.

### MHC class II

As opposed to MHC class I binding, which is mostly limited to peptides of similar length, the MHC class II molecule interacts with peptides of a wide length distribution and high compositional diversity [28]. Binding of a peptide to an MHC class II molecule is primarily determined by a core of normally 9 amino acids, but the composition of the regions flanking the binding core (peptide flanking region, PFR) has been shown to also affect the binding strength of a peptide [29], [30]. Identifying the binding motif and binding register for MHC class II binding peptides is thus a problem that inherently requires simultaneous alignment and binding affinity identification. Here, an MHC class II benchmarking was obtained from the recent publication by Wang et al. [25]. The performance was estimated for each allele using a 5 fold cross validation, where at each step 4/5 of the data were used to train the neural networks, and 1/5 were left out for evaluation. For cross-validation, we preserved the same data partitioning as used in the original publication. In *Table 1*, the performance of *NNAlign* on the Wang set is compared to other publicly available methods for MHC class II prediction. These include SMM-align [19], ProPred/Tepitope [31], [32], as well as the original version of the *NN-align* algorithm [20]. The *NN-align*-based methods outperform their competitors on all alleles, confirming the ability of the neural networks in dealing with alignment problems. The difference with the original *NN-align* method, which is due to differences in network architecture, is small and not significant (p>0.2, binomial test). For this example involving unaligned data, the *NNAlign* server competes with comparable state-of-the-art methods.

### Choosing the optimal motif length

**Figure 2:**
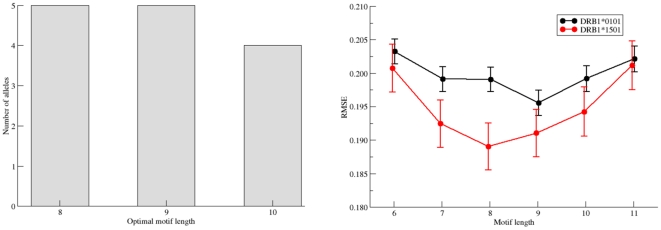
Identification of optimal motif length using the *NNAlign* method. **Left panel:** Histogram of the optimal motifs lengths for the 14 HLA-DR molecules in the Wang dataset as identified by the *NNAlign* method. Right panel: Predictive performance measured in terms of the root mean square error (RMSE) between observed and predicted values as a function of the motif length for the two molecules DRB1*0101 and DRB1*1501. *NNAlign* was trained using the same parameters settings described in Figure 4. At each motif length are shown the mean and standard error of the mean RMSE as estimated by bootstrap sampling. For DRB1*0101 a single consistent optimal motif length of 9 amino acids is found. For DRB1*1501 all motif length 8–11 had statistically indistinguishable performance (paired t-test).

Different positions in a binding motif can be more or less informative, and the ends of a motif can often not be clearly delineated. This prompts the question of how many positions are necessary and sufficient to represent a given motif and how the length of a motif is defined. *NNAlign* allows searching for the optimal motif length in a quantitative peptide data set. Here, the best motif length is the one that yields, in a cross-validation experiment, the lowest root mean square error (RMSE) between observed and predicted values. By this token, a terminal position is included in the motif if it contributes with information at a level above what could be considered to be noise. In contrast, if the inclusion of a putative terminal position does not lead to a reduction in the RSME then it can be concluded that it does not add useful parameters to the model; rather, it lowers the predictive performance and should be omitted. This approach was used to suggest the motif length of the 14 MHC class II HLA-DR alleles, which were searched for optimal predictive performance by scanning through possible lengths from 6 to 11 amino acids. *NNalign* will report the length associated with the lowest RMSE value as the optimal motif length (see Figure 2, left hand panel). Nonetheless, the user is advised to inspect the sequence logo as well as the performance plot of the RMSE as a function of the motif length to evaluate whether the dependence upon length appears significant. As defined here and illustrated in Figure 2 right panel, the 9-mer preference of HLA-DRB1*01:01 is significant, whereas the apparent 8-mer preference of HLA-DRB1*15:01 is not significant. In fact, for the 14 HLA-DR molecules included in the benchmark, only one was found to have a single consistent optimal motif length (DRB1*0101 with a motif length of 9 amino acids). For all other molecules the method did identify more than one possible optimal motif length. However, all motif lengths fell in the range of 7 to 10 amino acids, and in all cases a 9-mer motif was compatible with being the optimal motif length.

### Improving the LOGO sequence motif representation by an offset correction

**Figure 3:**
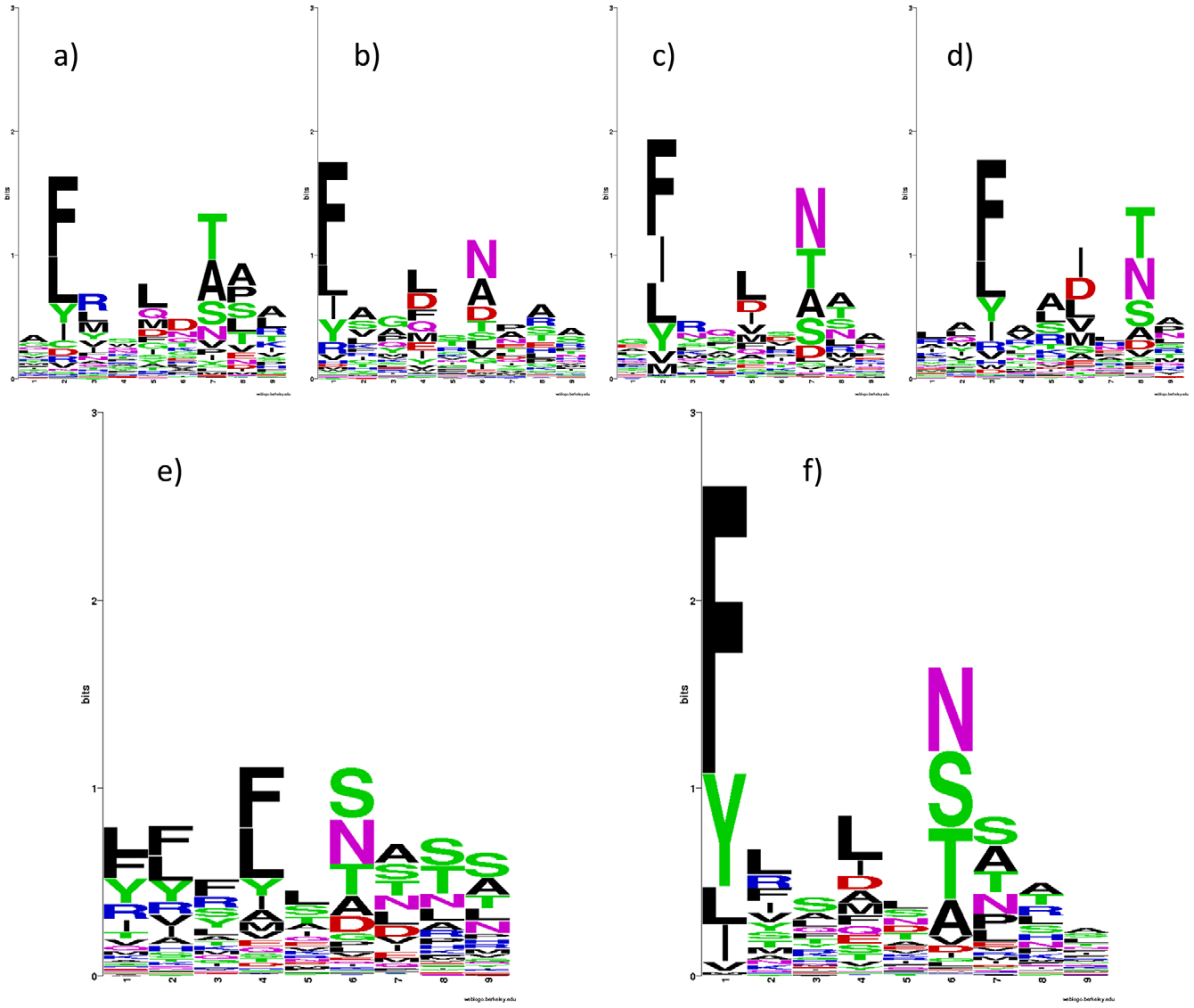
Sequence logos for HLA*DRB1-0401. In panels a) to d) are shown sequence logos for 4 single networks from the network ensemble created with *NNAlign*. The fundamental pattern appears in all these networks, but they place the anchors at different position of the core. e) shows the core of the 20 networks ensemble without offset correction; in f) offset correction was used to realign the logos to a common register.

In order to enhance predictive performance, the *NNAlign* method exploits an ensemble of neural networks [20], [33], which have been trained on different subsets of the data, and/or from alternative configurations of the network architecture (i.e. different number of hidden neurons and/or encoding schemes). As a consequence of different architectures and starting conditions, individual networks might disagree on the exact boundaries of the motif. This disagreement would complicate the visualization of the motif if this was represented as a simple overlay of the individual motifs as exemplified in Figure 3, where sequence logos for four different networks from the ensemble trained on HLA*DRB1-04:01 binding data are shown in panels A through D. The individual networks agree on identifying the same strong primary anchor residues and positions, however, each single network identifies different ends (i.e. suggests different registers of the same motif; *in casu* starting at positions 1, 2, 2 and 3 of the predicted nonamer peptide). The weak C-terminal primary anchor residue of HLA*DRB1-04:01 probably explains why the boundaries are difficult to determine. A simple overlay of the predictions from individual networks would result in a muddled motif as depicted in Figure 3, panel E. Implementing a Gibbs sampler approach, where matrix representations of the core motifs of different networks are aligned, we introduced an off-set correction for each network aiming at maximizing the information content of a combined logo representation of the motif. This approach led to a considerable improvement in the visual logo representation of the binding motif (Figure 3, panel F). Offset correction is included as an integral part of the method to enhance motif visualization.

### Characterizing the binding motif of HLA-DR molecules using the NNAlign method

**Figure 4:**
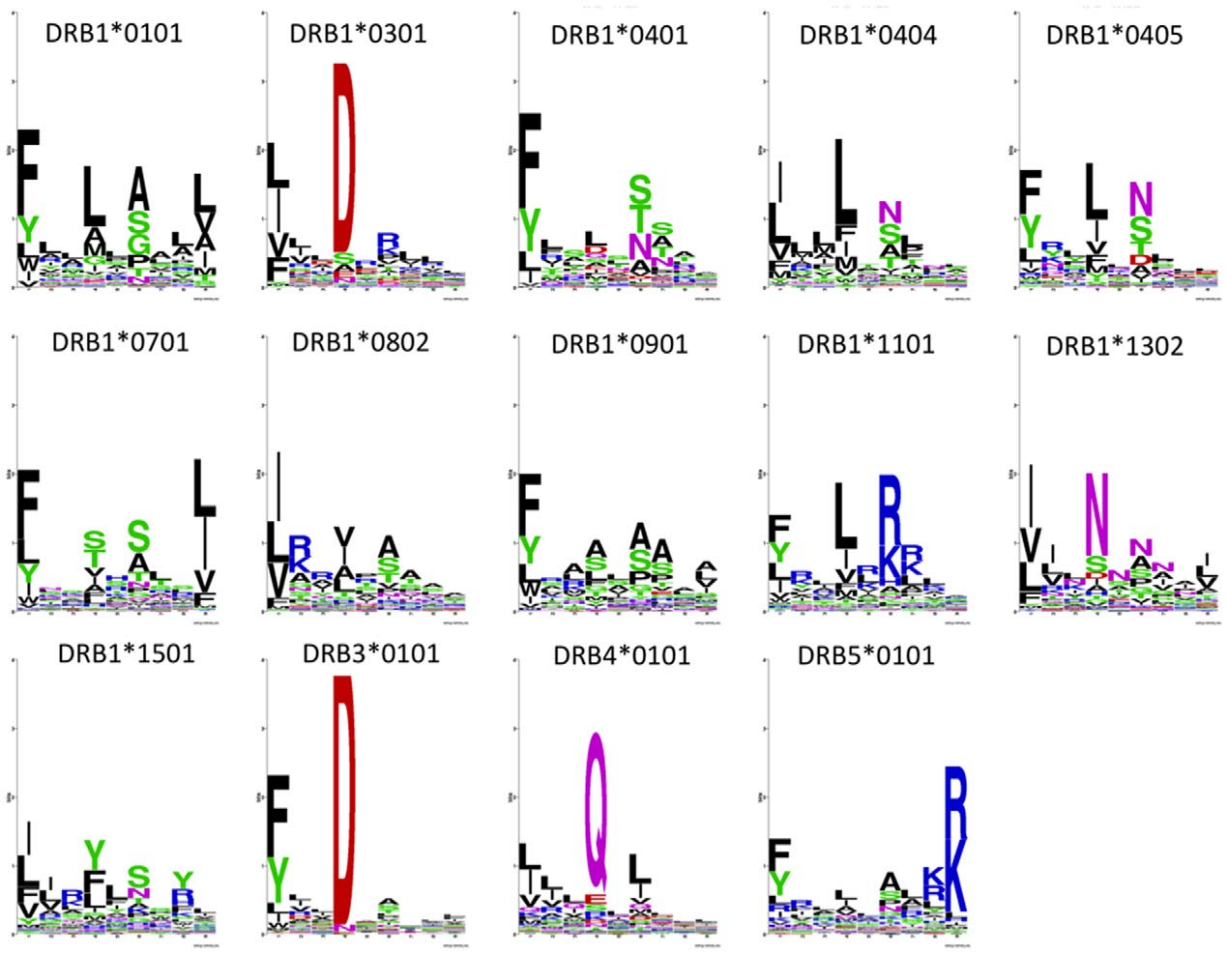
Sequence logo representation of the binding motifs for the 14 HLA-DR molecules contained in the Wang MHC class II data set. *NNAlign* was trained with Blosum encoding, including peptide length and flanking region length, PFRs of 3 amino acids, homology clustering at threshold 0.8 using all data points, 20 hidden neurons and a 5-fold cross-validation without stopping on the best test set performance.. Sequence logos are calculated as described in material and methods and visualized using the WebLogo program [48].

To illustrate the power of the *NNAlign* method to capture the binding motifs within unaligned quantitative peptide data, we applied the method to derive sequence logo representations of the 14 MHC class II HLA-DR molecules included the Wang dataset. *NNAlign* was trained with a binding motif length of 9 amino acids, Blosum encoding, including peptide length and flanking region length, and PFRs of 3 amino acids, homology clustering at threshold 0.8 using all data points, 20 hidden neurons and a 5-fold cross-validation without stopping at the best test set performance. These parameters were found to be optimal in the original NN-align paper for MHC class II binding prediction [20], with the only difference that here we choose a single value for hidden layer size for a matter of prediction speed. Individual networks are aligned to a common register using the offset correction strategy previously described. The sequence logos obtained are shown in Figure 4. The sequence logos reflect the overall consensus of the binding motifs for HLA-DR molecules, namely a prominent P1 anchor with strong amino acids preference towards hydrophobic amino acids in general, and aromatic amino acids as F and Y in particular, and the presence of two or more additional anchors at P4, P6 and/or P9 each with a unique amino acid preference. Even though most of these motifs exhibit a strong preference for hydrophobic and neutral amino acids at most anchor positions, some dramatic deviations from this general pattern exist. Examples of this are the motifs of DRB1*0301 and DRB1*1101 molecules that have strong preferences for charged amino acids at P4 and P6, respectively.

### Handling large data sets exemplified by protease recognition of high-density peptide microarrays

**Figure 5:**
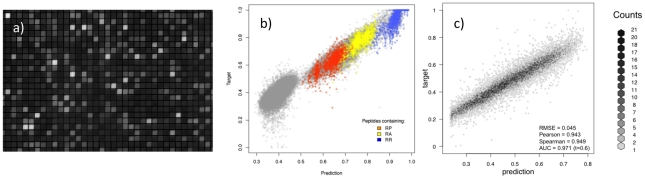
Analysing high-density peptide array data with *NNAlign*. a) Fluorescence microscopy picture of a peptide microarray. The image is a magnified segment of the peptide chip used in the trypsin cleavage analysis. b) Trypsin peptide-chip data. The normalized observed (target) likelihood of cleavage as a function of the prediction score for the trypsin data set. Localizations of peptides containing the pairs of amino acids RP, RA or RR are highlighted in the plot. Proline (P) is known to prevent cleavage after arginine (R), whereas cleavage is observed with other amino acids such as R and A. c) Chymotrypsin peptide-chip data. Correlation plot between predicted and measured (target) data from the chymotrypsin data set. Values are binned by their x,y proximity, so that the scatterplot represents the density of data in each bin. *NNAlign* was trained with linear rescaling of the quantitative data, a motif length of 4 amino acids without inclusion of PFR encoding, Blosum encoding of peptide sequences, a combination of 3,7,15 hidden neurons, 10 initial seeds, 5-fold exhaustive cross-validation, training was stopped on the best test set performance.

A peptide microarray containing a total of >100,000 peptides (49,838 of which were unique) was digested with the protease trypsin. The peptide sequences had been synthesized using the theme Ac-GAGAXXXXXGAGA, where Ac- is acetyl blocking the peptide alpha-amino group prior to digestion, and X represents amino acids chosen randomly from the 20 natural amino acids (except lysine, as this residue contains an epsilon-amino group, which even without digestion would be detectable (see Materials and Methods for details)). As a result, free amino groups can only be expressed by trypsin cleaved peptides, which then can then be labeled with Dylight549 and quantitated by fluorescence microscopy. A fluorescence microscopy picture of such a digested and stained peptide microarray (Figure 5a) demonstrates both the resolution of the photolithographic peptide synthesis strategy and the dynamic range of the free amino group detection strategy. The resulting data was log-transformed and rescaled to obtain a data distribution covering the spectrum between 0 and 1, which - along with the corresponding peptide sequences encoded as Blosum scores without flanking regions - were used to train an *NNAlign* method. Training was done with a motif length of 5, a fixed number of 3 hidden neurons, 5-fold exhaustive validation, and stopping at the best test set performance. The prediction method yielded a Pearson correlation between measured values and predictions of r = 0.971, a Spearman correlation of ρ = 0.910, and receiver operating characteristics (ROC) area under the curve (AUC) of 0.997 (using a target threshold of t = 0.5). The very high performance measures of the resulting *NNAlign* method demonstrate both that the recorded peptide digestion data contains a consistent and intelligible signal, and that the *NNAlign* method is capable of deciphering and predicting this extraordinary large number of sequence-dependent peptide signals. The correlation scatterplot feature of the *NNAlign* web-server output, which compares predicted vs. observed values, further supports the validity of both the peptide microarray and of the *NNAlign* method. The correlation scatterplot for the trypsin digestion data reveals two major populations of peptides, one composed of non-degradable, non-predicted peptides and one containing weakly to strongly degradable, predicted peptides (Figure 5b). Few (0.7%) of the former peptides contained Arginine, whereas most (97.1%) of latter peptides contained Arginine. This is exactly what one would have expected from a peptide digestion with trypsin, which is known to cleave at the C-terminal side of amino acids Arginine (and Lysine, which has been excluded here, see above) [34]. For illustration purposes, Figure 5b includes a color-enhanced visualization of certain dipeptide sequences (note, this is not a standard feature of the *NNAlign* server) showing that RP sequences are resistant, RA sequences are quite susceptible, and RR sequences appear extremely susceptible to trypsin digestion. Thus, the known trypsin resistance of RP sequences is both demonstrated by the peptide microarray and subsequently captured by the *NNAlign* method. Note, that both the peptide microarray and the *NNAlign* generate a continuous set of measurements and predictions showing that trypsin cleavage involves a more complex interaction than a simple recognition solely of an Arginine residue (and by inference a Lysine residue), which would have resulted in a cleaved/non-cleaved classification [35]. It is also important to note that the detection strategy employed here does not reveal where the protease cleavage has occurred, but merely that the protease has recognized the peptide as a substrate and cleaved it somewhere.

A similar high-density peptide microarray driven approach was next used to address the specificity of the protease chymotrypsin, which is known to preferentially cleave at the C-terminal of tyrosine, phenylalanine and tryptophan (albeit not if followed by a proline). A high-density peptide microarray containing about 50,000 peptides (16,526 unique peptides) was generated according to the theme Ac-GAGAXXXXGAGA, treated with chymotrypsin, labeled with TAMRA and quantitated by fluorescence microscopy. The resulting data was used to train an *NNAlign* method (using the settings described in Figure 5). The correlation scatterplot of the measured versus predicted values exhibits a very strong linear correlation with a Pearson of r = 0.943 demonstrating that the peptide microarray data contains a consistent signal that reliably has been captured by the *NNAlign* method.

## Discussion

The amount of data deposited in genomic and proteomic databases has been growing exponentially for many years [36]. Due to recent technological advances that have enabled whole-genome sequencing and made whole-proteome analysis a realistic goal, sequence data will accumulate at an even faster pace in the future where single laboratories, even single experiments, can generate data at the “omics” level. This is amply illustrated here where a high-density peptide microarray technology allowed the parallel synthesis of more than 100,000 discrete peptide sequences per array, and the collection of a corresponding number of quantitative peptide-receptor interaction data - all within a single experiment.

The biggest hurdle of future “omics” research may easily become that of making sense of such large-scale biologic sequence data [37]. Presently, the “omics” experimentalist requires assistance from specialized and highly trained bioinformaticians capable of large-scale data handling and interpretation. Ideally, however, he or she should not only be armed with high-throughput data-generation technologies, but also with reasonably easy and robust bioinformatics methods allowing the experimentalist to analyze his or her own data. This would permit an immediate analysis of experimental results and assist in rational designs of next generation experiments aimed at extending the original analysis e.g. providing *in silico* tools for searches that potentially could encompass entire proteomes. Enabling the same person to do large-scale experiments and analysis should result in a better integration between design, experiment, and interpretation – and eventually support the development of new hypotheses. Unfortunately, suitable bioinformatics resources aimed at the non-expert user are currently scarce, and rarely web-based. In our experience, open source software packages such as Weka [38] are not capable of performing concurrent alignment and motif identification, and are not suited for treating large-scale data sets. A widely used method for motif discovery, MEME [39], can perform searches for un-gapped sequence patterns in DNA or protein sequences, and offers a user-friendly online server to the untrained user. However, this method is not designed for use in quantitative data, such as peptide-MHC binding or peptide microarray data.

To the best of our knowledge, *NNAlign* is the first web-based bioinformatics solution that allows non-expert users to discover short sequence motifs in quantitative peptide data. As shown here, *NNAlign* easily competes with state-of-the-art methods for identifying peptide-binding motifs of aligned (exemplified by MHC class I) as well as unaligned (exemplified by MHC class II) quantitative peptide sequence data. Further, demonstrating the general utility of *NNAlign*, we have used it to characterize the cleavage specificities of proteases from high-throughput peptide array data. If a sufficient number of training examples can be generated, including negative instances, we could envision applying the method also on data generated by phage display peptide libraries. Other instances of recognition of short specific peptide motifs occurs frequently in biology where they are involved in molecular interaction, recognition, signaling, internalization, modification etc (e.g. phosphorylation, dephosphorylation, trafficking motifs, SH2 and SH3 domains, glycosylation, lipidation, etc. In contrast to domain recognition, short linear peptide sequences are thought to be particularly difficult to identify due to their unordered structure [40]. *NNAlign* appears to be ideally suited to identify such short linear peptide targets. Due to its simple interface and robust performance, we believe the method to constitute a significant tool providing the non-bioinformatician end-user with the ability to perform advanced bioinformatics-driven analysis of large-scale peptide data sets.

## Materials and Methods

### MHC class I data set

The data set of quantitative peptide-MHC class I binding affinity data published by Peters et al. [27] contains data from 48 different human, mouse, macaque and chimpanzee alleles. We selected 12 representative human alleles, and extracted binding data for 9-mer peptides maintaining the subsets of the original benchmark. This allows comparing the performance of *NNAlign* to the other methods presented in the paper by Peters et al.

### MHC class II data set

A large set of over 17,000 HLA-peptide binding affinities was published by Wang et al. [25] containing data from several different human alleles including HLA DR, DP and DQ alleles. For each allele, the predictive performance of various methods was estimated on the similarity reduced (SR) data set, where sequence similarity is minimized in order to avoid overlap between cross-validation subsets. We preserved the same subsets for our cross-validation, for easy comparison of the results and predictive performances.

### Peptide arrays

Peptide arrays were synthesized by Schafer-N, Copenhagen, Denmark using a maskless photolithographic technique [41] in which 365 nm light is projected onto NPPOC-photoprotected [42], [43] amino groups on a glass surface in patterns corresponding to the synthesis fields. Details of the technique will be published elsewhere, but briefly, the patterns were generated using digital micromirrors and projected onto the synthesis surface using UV-imaging optics. In each layer of amino acids, the relevant amino acids were coupled successively to predefined fields after UV-induced removal of the photoprotection groups in those fields. The couplings were made using standard Fmoc-amino acids activated with HBTU/DIEA in NMP. After coupling of the last Fmoc-amino acid in each layer, all Fmoc-groups were removed in 20% piperidine in NMP and replaced by NPPOC groups coupled as the chloroformate in DCM with 0.1 M DIEA. The procedure was then repeated until all amino acids had been added to the growing peptide chains. Final cleavage of side protection groups was performed in TFA∶1,2-ethanedithiol∶water 94∶2∶4 v/v/v for 2 h at room temperature.

#### Trypsin data

Peptide arrays were incubated for 30 min at room temperature with 0.1 g/L bovine Trypsin (Sigma T9201) dissolved in 0.1 M Tris/Acetate pH 8.0. After washing in the same buffer containing 0.1% SDS, the slides were washed with deionized water and air-dried. Staining of amino groups exposed by enzyme cleavage was made by incubation the slide for 30 min in 0.1 mg/mL Dylight549-NHS (Thermo Scientific) in 9∶1 v/v n-methyl pyrrolidone∶0.1M n-methyl morpholine/HCl pH 8 for 10 minutes.

#### Chymotrypsin

Peptide arrays were incubated for 30 min at room temperature with 0.1 g/L bovine Chymotrypsin (Sigma C4129) dissolved in 0.1 M Tris/Acetate pH 8.0. After washing in the same buffer containing 0.1% SDS, the slides were washed with deionized water and air-dried. Staining of amino groups exposed by enzyme cleavage was made by incubation of the slides for 10 min in 1 mM 5(6)-TAMRA (carboxytetramethylrhodamine, Fluka 21953) activated with 1 eq HBTU, 2 eq DIEA in n-methylpyrrolidone.

#### Recording of signals from peptide arrays

After incubation with activated fluorochromes, the peptide array slides were washed in the incubation buffer without fluorochrome followed by washings in n-methylpyrrolidone and dichloromethane and air-dried. Images of the arrays were recorded using a MVX10 microscope equipped with a MT10_D fluorescence illumination system and a XM10 CCD camera (all from Olympus). The excitation wavelength was 530–550 nm and the emission filter was 575–625 nm. The images were analyzed using the PepArray analysis program (Schafer-N, Copenhagen Denmark).

### The *NNAlign* Web Server

#### Data pre-processing

The quantitative peptide data entered by the user is rescaled to be between 0 and 1 before being fed to the neural network. The user is also given the option to apply a logarithmic transformation to the raw data, if its distribution appears to be too squashed towards low values. Outliers deviating more than 3 standard deviations from the average, which after rescaling would produce sparse regions in the spectrum with no data, are set at a value of exactly 3 standard deviations. This procedure produces ideal data for artificial neural network (ANN) training, with all values in the range [0∶1] and the bulk of the data in the central region of the spectrum. The parameters for the rescaling function are defined separately on each of the training sets used in cross-validation, and then also applied to rescale their relative test sets.

#### Subsets for cross-validation

In a *n*-fold cross-validation, *n* subsets are created from the complete dataset, and at each step *n-1* subsets are used for training and 1 subset for testing. *NNAlign* offers three alternatives to create the subsets: i) random, splits the data into *n* subsets randomly; ii) homology clustering, uses a Hobohm 1 algorithm [44] to identify sequences that share an ungapped alignment with more than a specified fraction of matches; iii) common motif clustering, looks for stretches of identical amino acid between pairs of sequences as described by Nielsen et al. [19]. For both methods ii) and iii) similar sequences are grouped together in the same subset, but it is possible to choose to only include one representative for each group and disregard the other sequences from training. In this phase, if the input data contains repeated flanks (as might be the case in peptide array experiments, where linker sequences can be attached at the extremities of all peptides), these flanks are discarded, as they would affect the overlap estimation. If the user reckons that the repeated flanks might contain meaningful biological signal, an option allows retaining them in the training data. Note that in common motif clustering, the motif length is taken as the smallest in the interval of length given by the user. Thus, depending on the selected interval the subsets might be constructed in a different way and that could influence the cross-validated performance.

#### Neural network training

The neural network training is performed as described by Nielsen et al. [20]. Initially, all network weights are assigned random values. From the current network configuration, the method selects the optimal n-mer core (and potential peptide flanking residues) for each of the peptides within the training set. The network weights are next updated, to lower the sum of squared errors between the observed and predicted score, the cores are redefined based on the new network configuration, and the procedure is iterated.

An ensemble of ANNs is trained on the cross-validation subsets, with architecture parameters specified by the user. The motif length, encoding of flanks and peptide length determine the size of the input layer. If the motif length is given as an interval of values, multiple runs of ANN training are performed on the different lengths, and the length that produces the best cross-validated performance in terms of root mean square error (RMSE) is chosen for the final ensemble. The number of hidden neurons may be specified as a list of multiple values, so that an ensemble of networks is constructed with hidden layers of different sizes. Each architecture is trained multiple times, starting from different initial random configurations, to avoid as much as possible choosing sub-optimal solutions producing local minima. Sequences can be presented to the network either with Sparse or Blosum encoding. In Sparse encoding, a vector of length N represents each amino acid, where all values are identical apart from the one representing the observed amino acid. Blosum encoding, on the other hand, takes into account amino acids similarity and partially allows substitutions of similar amino acids while penalizing very dissimilar ones [45].

#### Performance measures

Cross-validation allows estimating a method performance without the need of external evaluation data. The subsets reserved as test-sets are run through the network trained in the same cross-validation step, and Pearson's correlation, RMSE and Spearman correlation are calculated between observed and predicted values.

It is possible to use the internal subsets to stop the training phase on the best test set performance in terms of RMSE. In this mode, performance can be estimated in an exhaustive or in a fast way. Exhaustive *n*-fold cross-validation (CV) consists of a nested CV procedure. At each step, 1 subset is left out as evaluation set, and the remaining subsets are used to generate a network ensemble in an *n-1* CV training. In this CV training, the selected network configuration is the one that gives the minimum RMSE on the stopping set. Next the predictions for the evaluation data are estimated as a simple average of the prediction values for each network in the training ensemble. The exhaustive CV procedure adds one level to the cross-validation and increases greatly the running time. In alternative, the fast evaluation skips one nested level by using the same subset for stopping and evaluating performance, for a quicker but likely less accurate performance estimation.

#### Final network ensemble

With cross-validated ANN training, each network has been evaluated on data not included in the training. The networks can then be ranked by performance, and only the top *N* for each cross-validation step will be included in the final ensemble, with *N* specified by the user. The final network ensemble can be downloaded to local disk, and used for predictions on new data by loading it to the *NNAlign* server submission page.

#### Sequence motif logo

A list of 100,000 random naturally occurring peptides with length L = motif length+2 * flank length, generated from random UniProt [46] sequences, is presented to the individual networks in the ensemble. For each network, the 1% peptides that obtain the highest prediction scores are used to create a position specific scoring matrix (PSSM) that represents the motif captures by the neural network. Using a Gibbs sampler approach, all PSSMs are aligned to maximize the information content of the combined matrix. This “offset correction” step is obtained by repeatedly attempting to shift the starting position of randomly chosen PSSMs, and accepting/rejecting the move according to the conventional Metropolis Monte Carlo probability relation [47]:
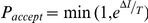
Where ΔI is the change in information content between the new and old offset configuration and T is a scalar that is lowered during the calculation. The process assigns to each PSSM, and to its relative network, an offset value that quantifies the shift distance from other networks. The re-aligned cores from the 1% scoring of 100,000 peptides are finally used to generate a combined sequence logo with the WebLogo program [48]. The offset correction can be skipped if the user chooses to, and in this case the logo is simply created by presenting the list of random peptides to the ANN final ensemble and selecting the 1% peptides that obtain the overall best score.

#### Evaluation data

Additional data not included in the training can be uploaded to the *NNAlign* Server as an evaluation set. Evaluation data must be a list of peptides, with or without associated values, or a file in FASTA format. In the first case, all peptides are run through the trained network ensemble, and scored accordingly to their best alignment core. If values are provided together with peptides, they are assumed to be target values for validation purposes, and statistical measures between these values and predictions are calculated. In the case a FASTA file is loaded as evaluation set, the sequences therein contained are cut into peptides of length L = motif length+2 * flank length, shifting the starting position of one amino acid at a time. The generated peptides are all fed to the network to identify those that most closely match the motif learned by the ANNs. The results are sorted by prediction value, so that the best candidates are displayed at the top of the list.

### Making sequence logos

Sequence logos were introduced by Schneider et al. [49] as a way to represent graphically the pattern in a set of aligned sequences. The height *R_i_* of each column *i* in the logo is given as the information content in bits of the alignment at that particular position, and for a sufficiently large number of sequences and a 20-letter alphabet it is calculated as:
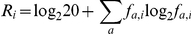
where *f_a,I_* is the frequency of amino acid *a* at position *i*. The relative height *h_a,i_* of amino acid *a* at position *i* is:
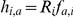
The value of *R_i_* varies between 0, for a position with maximum entropy, to log_2_20, for a completely conserved position in the alignment. Thus, the height of a column in the sequence logo indicates the importance of a certain position in defining the motif, and the height of each letter in the column the amino acid preference at that position. Amino acid letters are colored according to their chemical properties: polar amino acids (C, G, S, T, Y) are shown in green and (N, Q) pink, basic (K. R, H) in blue, acidic (D, E) in red, and hydrophobic (A, L, I, V, F, M, P, W) in black.

## References

[1] James W (2001). Nucleic acid and polypeptide aptamers: a powerful approach to ligand discovery.. Curr Opin Pharmacol.

[2] Hoppe-Seyler F, Crnkovic-Mertens I, Tomai E, Butz K (2004). Peptide aptamers: specific inhibitors of protein function.. Curr Mol Med.

[3] Lin J, Bardina L, Shreffler WG, Andreae DA, Ge Y (2009). Development of a novel peptide microarray for large-scale epitope mapping of food allergens.. J Allergy Clin Immunol.

[4] Schutkowski M, Reineke U, Reimer U (2005). Peptide arrays for kinase profiling.. Chembiochem.

[5] Han X, Yamanouchi G, Mori T, Kang JH, Niidome T (2009). Monitoring protein kinase activity in cell lysates using a high-density peptide microarray.. J Biomol Screen.

[6] Halperin RF, Stafford P, Johnston SA (2011). Exploring antibody recognition of sequence space through random-sequence peptide microarrays.. Mol Cell Proteomics.

[7] Masch A, Zerweck J, Reimer U, Wenschuh H, Schutkowski M (2010). Antibody signatures defined by high-content peptide microarray analysis.. Methods Mol Biol.

[8] Uttamchandani M, Yao SQ (2008). Peptide microarrays: next generation biochips for detection, diagnostics and high-throughput screening.. Curr Pharm Des.

[9] Christensen JK, Lamberth K, Nielsen M, Lundegaard C, Worning P (2003). Selecting informative data for developing peptide-MHC binding predictors using a query by committee approach.. Neural Comput.

[10] Bendtsen JD, Nielsen H, von Heijne G, Brunak S (2004). Improved prediction of signal peptides: SignalP 3.0.. J Mol Biol.

[11] Juncker AS, Willenbrock H, Von Heijne G, Brunak S, Nielsen H (2003). Prediction of lipoprotein signal peptides in Gram-negative bacteria.. Protein Sci.

[12] Kim JH, Lee J, Oh B, Kimm K, Koh I (2004). Prediction of phosphorylation sites using SVMs.. Bioinformatics.

[13] Blom N, Gammeltoft S, Brunak S (1999). Sequence and structure-based prediction of eukaryotic protein phosphorylation sites.. J Mol Biol.

[14] Obenauer JC, Cantley LC, Yaffe MB (2003). Scansite 2.0: Proteome-wide prediction of cell signaling interactions using short sequence motifs.. Nucleic Acids Res.

[15] Schwartz D, Gygi SP (2005). An iterative statistical approach to the identification of protein phoshorylation motifs from large-scale data sets.. PLoS Comput Biol.

[16] Lundegaard C, Lund O, Buus S, Nielsen M (2010). Major histocompatibility complex class I binding predictions as a tool in epitope discovery.. Immunology.

[17] Nielsen M, Lundegaard C, Worning P, Hvid CS, Lamberth K (2004). Improved prediction of MHC class I and class II epitopes using a novel Gibbs sampling approach.. Bioinformatics.

[18] Bhasin M, Raghava GP (2004). SVM based method for predicting HLA-DRB1*0401 binding peptides in an antigen sequence.. Bioinformatics.

[19] Nielsen M, Lundegaard C, Lund O (2007). Prediction of MHC class II binding affinity using SMM-align, a novel stabilization matrix alignment method.. BMC Bioinformatics.

[20] Nielsen M, Lund O (2009). NN-align. An artificial neural network-based alignment algorithm for MHC class II peptide binding prediction.. BMC Bioinformatics.

[21] Nielsen M, Lund O, Buus S, Lundegaard C (2010). MHC Class II epitope predictive algorithms.. Immunology.

[22] Castelletti D, Fracasso G, Righetti S, Tridente G, Schnell R (2004). A dominant linear B-cell epitope of ricin A-chain is the target of a neutralizing antibody response in Hodgkin's lymphoma patients treated with an anti-CD25 immunotoxin.. Clin Exp Immunol.

[23] Hua R, Zhou Y, Wang Y, Hua Y, Tong G (2004). Identification of two antigenic epitopes on SARS-CoV spike protein.. Biochem Biophys Res Commun.

[24] Sompuram SR, Vani K, Hafer LJ, Bogen SA (2006). Antibodies immunoreactive with formalin-fixed tissue antigens recognize linear protein epitopes.. Am J Clin Pathol.

[25] Wang P, Sidney J, Kim Y, Sette A, Lund O (2010). Peptide binding predictions for HLA DR, DP and DQ molecules.. BMC Bioinformatics.

[26] Rao X, Costa AI, van Baarle D, Kesmir C (2009). A comparative study of HLA binding affinity and ligand diversity: implications for generating immunodominant CD8+ T cell responses.. J Immunol.

[27] Peters B, Bui HH, Frankild S, Nielson M, Lundegaard C (2006). A community resource benchmarking predictions of peptide binding to MHC-I molecules.. PLoS Comput Biol.

[28] Rammensee H, Bachmann J, Emmerich NP, Bachor OA, Stevanovic S (1999). SYFPEITHI: database for MHC ligands and peptide motifs.. Immunogenetics.

[29] Lovitch SB, Pu Z, Unanue ER (2006). Amino-terminal flanking residues determine the conformation of a peptide-class II MHC complex.. J Immunol.

[30] Godkin AJ, Smith KJ, Willis A, Tejada-Simon MV, Zhang J (2001). Naturally processed HLA class II peptides reveal highly conserved immunogenic flanking region sequence preferences that reflect antigen processing rather than peptide-MHC interactions.. J Immunol.

[31] Singh H, Raghava GP (2001). ProPred: prediction of HLA-DR binding sites.. Bioinformatics.

[32] Sturniolo T, Bono E, Ding J, Raddrizzani L, Tuereci O (1999). Generation of tissue-specific and promiscuous HLA ligand databases using DNA microarrays and virtual HLA class II matrices.. Nat Biotechnol.

[33] Petersen TN, Lundegaard C, Nielsen M, Bohr H, Bohr J (2000). Prediction of protein secondary structure at 80% accuracy.. Proteins.

[34] Olsen JV, Ong SE, Mann M (2004). Trypsin cleaves exclusively C-terminal to arginine and lysine residues.. Mol Cell Proteomics.

[35] Schilling O, Huesgen PF, Barre O, Auf dem Keller U, Overall CM (2011). Characterization of the prime and non-prime active site specificities of proteases by proteome-derived peptide libraries and tandem mass spectrometry.. Nature protocols.

[36] Lathe W, Williams J, Mangan M, Karolchik D (2008). Genomic data resources: challenges and promises.. Nat Edu.

[37] Nilsson T, Mann M, Aebersold R, Yates JR, Bairoch A (2010). Mass spectrometry in high-throughput proteomics: ready for the big time.. Nature methods.

[38] Frank E, Hall M, Trigg L, Holmes G, Witten IH (2004). Data mining in bioinformatics using Weka.. Bioinformatics.

[39] Bailey TL, Williams N, Misleh C, Li WW (2006). MEME: discovering and analyzing DNA and protein sequence motifs.. Nucleic Acids Res.

[40] Neduva V, Russell RB (2006). DILIMOT: discovery of linear motifs in proteins.. Nucleic Acids Res.

[41] Singh-Gasson S, Green RD, Yue Y, Nelson C, Blattner F (1999). Maskless fabrication of light-directed oligonucleotide microarrays using a digital micromirror array.. Nat Biotechnol.

[42] Hasan A, Stengele KP, Giegrich H, Cornwell P, Isham KR (1997). Photolabile protecting groups for nucleosides: Synthesis and photodeprotection rates.. Tetrahedron.

[43] Bhushan KR, DeLisi C, Laursen RA (2003). Synthesis of photolabile 2-(2-nitrophenyl)propyloxycarbonyl protected amino acids.. Tetrahedron Letters.

[44] Hobohm U, Scharf M, Schneider R, Sander C (1992). Selection of representative protein data sets.. Protein Sci.

[45] Henikoff S, Henikoff JG (1992). Amino acid substitution matrices from protein blocks.. Proc Natl Acad Sci USA.

[46] UniProt (2008). The universal protein resource (UniProt).. Nucleic Acids Res.

[47] Metropolis N, Rosenbluth AW, Teller AH, Teller E (1953). Equation of State Calculation by Fast Computing Machines.. J Chem Phys.

[48] Crooks GE, Hon G, Chandonia JM, Brenner SE (2004). WebLogo: a sequence logo generator.. Genome Res.

[49] Schneider TD, Stephens RM (1990). Sequence logos: a new way to display consensus sequences.. Nucleic Acids Res.

